# Interactive Marine Spatial Planning: Siting Tidal Energy Arrays around the Mull of Kintyre

**DOI:** 10.1371/journal.pone.0030031

**Published:** 2012-01-11

**Authors:** Karen A. Alexander, Ron Janssen, Gustavo Arciniegas, Timothy G. O'Higgins, Tessa Eikelboom, Thomas A. Wilding

**Affiliations:** 1 Scottish Association for Marine Science, Scottish Marine Institute, Oban, Argyll, United Kingdom; 2 Institute for Environmental Studies, Vrije Universiteit, Amsterdam, The Netherlands; University of Otago, New Zealand

## Abstract

The rapid development of the offshore renewable energy sector has led to an increased requirement for Marine Spatial Planning (MSP) and, increasingly, this is carried out in the context of the ‘ecosystem approach’ (EA) to management. We demonstrate a novel method to facilitate implementation of the EA. Using a real-time interactive mapping device (touch-table) and stakeholder workshops we gathered data and facilitated negotiation of spatial trade-offs at a potential site for tidal renewable energy off the Mull of Kintyre (Scotland). Conflicts between the interests of tidal energy developers and commercial and recreational users of the area were identified, and use preferences and concerns of stakeholders were highlighted. Social, cultural and spatial issues associated with conversion of common pool to private resource were also revealed. The method identified important gaps in existing spatial data and helped to fill these through interactive user inputs. The workshops developed a degree of consensus between conflicting users on the best areas for potential development suggesting that this approach should be adopted during MSP.

## Introduction

Continuing decline in the environmental quality of many of the world's oceans as a consequence of human activities [1], [2], [3] has led to rethinking our strategies for management of the marine environment. The recognition that human activities occur within, and are dependent on, the ecosystem that supports them [4], has led to the concept of the Ecosystem Approach (EA) to management. While there is no consensus on the definition of the EA, certain characteristics are identifiable: a multi-sectoral focus, inclusion of ecosystem services within the decision making process and a recognition that human and ecological systems are tightly coupled [5]. Although the concepts of the EA have rapidly gained currency across the disciplines of natural and social sciences, as well as incorporation into the legal frameworks for ocean management, very few examples of the EA in the marine environment can be found.

There are three reasons for the gap between EA theory and marine management in practice. Firstly, offshore activities have traditionally been controlled by central government [6] where space is allocated within individual economic sectors rather than integrated between sectors [7]. This results in a lack of relevant multidisciplinary expertise. Secondly, spatial and value data are a prerequisite for the EA [8] and while ecosystem services may be identified their values are often not quantified or are unquantifiable, or are of inadequate spatial resolution to implement informed planning [9], [10]. Thirdly, public recognition of the tight coupling between ecological and social systems is not widespread. There is therefore a need to build capacity to understand and participate in the EA process.

In the EU the Marine Strategy Framework Directive (MSFD) mandates achieving Good Environmental Status (GEnS) in Europe's regional seas by 2020 through implementation of the EA [11]. To achieve GEnS, eight programmes of measures have been proposed which include: ‘communication, stakeholder involvement and raising public awareness’ and ‘spatial and temporal distribution controls: management measures which influence where and when an activity is allowed to occur’ (of which Marine Spatial Planning (MSP) is one example [7]) [12]. In parallel with achieving GEnS there is an increasing demand from competing activities for the use of marine space. In particular, support for the renewable energy sector has increased amidst concerns about energy security [13], economic competitiveness, regional development and the need to reduce greenhouse gasses [14] leading to an EU wide target for 20% energy to be provided by renewable energy by 2020 [15]. Much of the world's coastline is already committed meaning that the marine renewable energy industry is yet another user of marine space which may adversely impact existing sea users such as shipping and fishing [16].

Conflict is common in the sea, both between and within sectors [17]. It may arise during marine planning due to hidden differences in terms of needs and aspirations of divergent groups [18]. Conflict may also arise from a lack of consultation, with severe repercussions. During the development of a wind farm in the Languedoc-Roussillon region of France, a lack of publicly available information led to resentment and a judicial appeal against the building permit. When turbines were raised unannounced in the Rheinland-Pfalz region of Germany lawsuits led to the delay of planning and increased costs to the planners [19]. Furthermore, in South Africa, the absence of engagement or consultation with fishers led to illegal activity and aggressive incidents between fishers and law enforcement officials [20]. An inevitable increase in conflict between those competing for sea space, given the inevitable inclusion of offshore energy, means that tools and approaches which can alleviate such conflicts are necessary.

Spatial decision support systems (SDSS), using environmental and social data and models, can contribute to the efficient exchange of information between experts, stakeholders and decision makers [21], enable scenario analysis [22] and potentially resolve user conflict. SDSS is an established method for resource management, where spatial data is presented to stakeholders as part of a negotiation process [23], [24]. The marine environment, however, poses unique problems; it is both data limited and viewed as a common resource. Spatial data for marine management has been collected using hard copy maps in workshop settings [25] and more recently using web-based systems [26], [27], [28]. However, there are few examples of natural resource-based SDSS which both digitally collect data and facilitate stakeholder negotiation in a workshop setting based upon these data, allowing resource users to both make themselves visible within the spatial environment and play an active role in the emerging EA to natural resource management. Those which do are predominantly terrestrial focusing on disaster management [29] and rural land planning [21], [30], [31].

Using offshore energy as a case study, the objectives of this research were: (i) to identify potential conflicts between users of sea space, (ii) to develop an approach to gather spatial information concerning user values at the small spatial scales relevant to local marine planning, and ultimately (iii) to test if this approach could effectively use spatial information to support negotiation (and reduce conflict) in a stakeholder workshop setting: a method which could benefit offshore industry developers in particular and marine spatial planners in general in facilitating the EA to marine management.

## Materials and Methods

### Study Area

**Figure 1:**
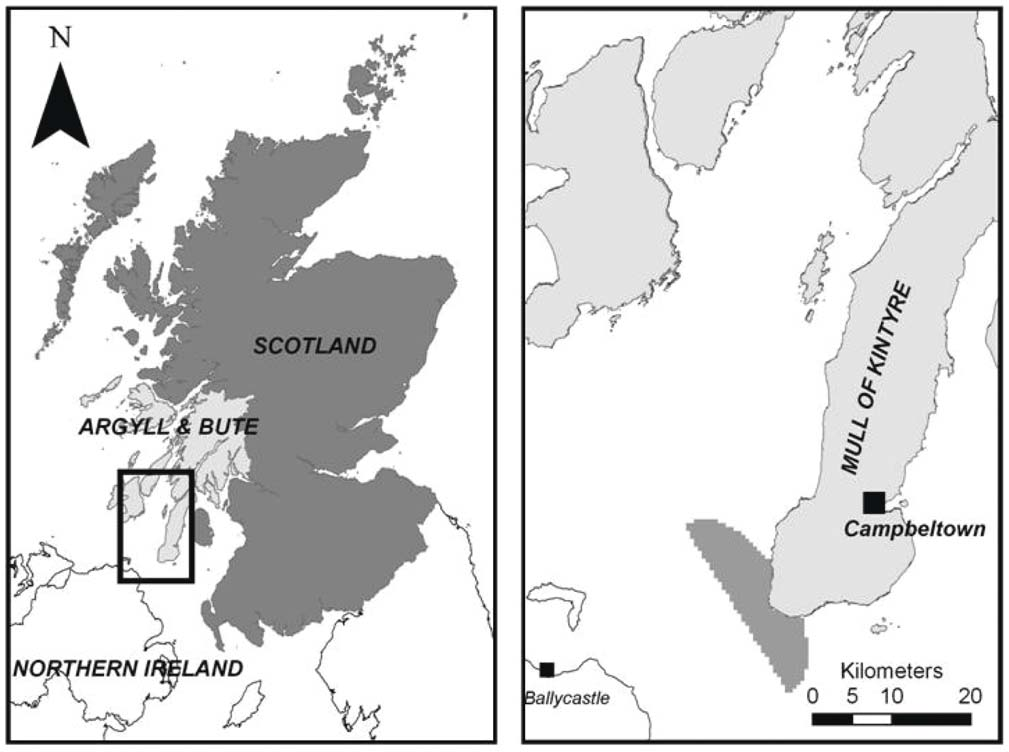
Location of study site.

Kintyre is a peninsula in south-west Argyll on the west coast of Scotland. The principal town is Campbeltown (∼9 km from the Mull, or tip of the peninsula, of Kintyre). The study site, off of the south-western tip of the Kintyre peninsula (Figure 1) is an area of proposed seabed lease offered by Marine Scotland and the Crown Estate (owners of the UK seabed) for the purpose of tidal energy development [32]. Diverse industries and activities operate within the site who may be affected by tidal energy development. In 2010 the announcement of the potential development of an offshore wind farm array at Machrihanish, in Kintyre, led to conflict within the community and between the community and the developers and to eventual abandonment of the project [33]. For this reason the Kintyre tidal energy lease site was chosen as the case study site for this research.

### Tools and equipment

Our study used an SDSS based on the method of Arciniegas et al. [34] which combined Geographic Information Systems (GIS), spatial Multi Criteria Analysis (MCA) and a touch-table to facilitate stakeholder dialogue in a workshop setting. GIS was used to present data, and spatial MCA provided a method with which to evaluate, compare, rank and present the performance of decision alternatives leading to a map-based ‘output’ from each of two workshops. The touch-table was the interface between the data and the workshop participants.

The touch-table (DiamondTouch table™), an interactive touch screen which allows simultaneous input from up to four users, was run concurrently with ArcGIS with the CommunityViz (http://www.communityviz.com/) extensions for interactive planning. Two tools were used in the workshops: (i) a ‘map valuation tool’ which allowed users to ‘draw’ onto the GIS display inputting features of importance or value which may not be on the original maps, and to change the value of these identified areas according to relative importance; (ii) a ‘MCA trade-off’ tool which used spatial MCA for comparison and ranking, making it possible to structure and aggregate the information to facilitate negotiation. The tool displays the ‘best’ and ‘worst’ areas (i.e. those with the highest and lowest values) for each stakeholder, allowing sea-uses to be compared and thus facilitating spatial trade-offs. A detailed description of the spatial MCA can be found in Arciniegas et al [34].

### Initial Data Processing

Stakeholder value maps to be used as a starting point for the stakeholder workshops were generated using GIS data sets from organisations including: Marine Scotland, British Ordnance Survey (EDINA), the Joint Nature Conservation Council (JNCC), the Royal Yachting Association (RYA), the Department for Energy and Climate Change (DECC) and Historic Scotland (for a full list of the original GIS data and their sources see Table S1). Using weighted summation, the data were aggregated by stakeholder group to generate stakeholder value maps. Weighted summation is a commonly-used method for spatial MCA [21], [35], [36]. First a score was standardized and multiplied by its weight. The weights represented the relative values of the criteria or objectives [37]. Weights were used at two levels: first to obtain a value score for each stakeholder; and second, to obtain aggregated values that could be used for negotiation. For example, the value for tidal devices was calculated as the weighted sum of: tidal flow, depth in meters, type of seabed, and distance to port. Weights at this level were set and specified using expert judgment. To calculate an overall value it was necessary to assign weights to each stakeholder. These weights are highly political and cannot be set by expert judgment. This study focused on the trade-offs between stakeholders and so the total value of a cell has little relevance for negotiation. Therefore no weights were assigned to the objectives of the stakeholders. Stakeholder specific values from 1–10 were assigned to a grid of 500 m×500 m cells based on the size of the study area and the likely size of tidal devices to be installed.

Six stakeholder maps were generated (i) tidal energy, (ii) commercial shipping, (iii) commercial fishing, (iv) recreational shipping, (v) tourism and (vi) environment. The aggregated maps were used as a basis for the first ‘local-knowledge’ workshop. Additional data collected at this workshop were added to the aggregated value maps for the second ‘negotiation’ workshop.

### Stakeholder Workshops

Two workshops were held in Campbeltown: (i) a ‘local-knowledge’ workshop and (ii) a ‘negotiation’ workshop. A list of the dates, locations and stakeholder representatives is given for each workshop in Table 1. Each workshop followed the same sequence of sessions; the first session described the research problem (planning for tidal energy deployment), how it would be addressed and the stakeholders involved; the second allowed participants to familiarise themselves with the tools, and the third involved participant use of the tools to fulfil the objectives of the research.

**Table 1:** Dates, topics and participants of workshop.

Dates	Locations	Topic	Representatives
3-May-11	Campbeltown, Argyll & Bute	‘Local Knowledge’ Workshop	Campbeltown Sub-Aqua Club; Campbeltown Sailing Club, local wildlife tour operator, local fisherman
5-May-11	Campbeltown, Argyll & Bute	‘Negotiation’ Workshop	Campbeltown Sailing Club; wildlife tour operator; local fisherman; Clyde Fisherman's Association; Scottish Renewables; Argyll & Bute Council

**Figure 2:**
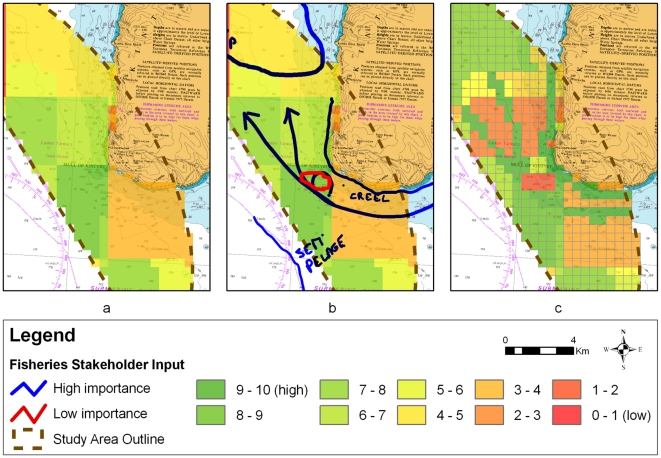
Steps taken during the ‘local knowledge’ workshop. a) the base value map, b) stakeholder drawings of areas of importance including a creel route, and an area of semi-pelagic fishing, c) the re-valued map.

**Figure 3:**
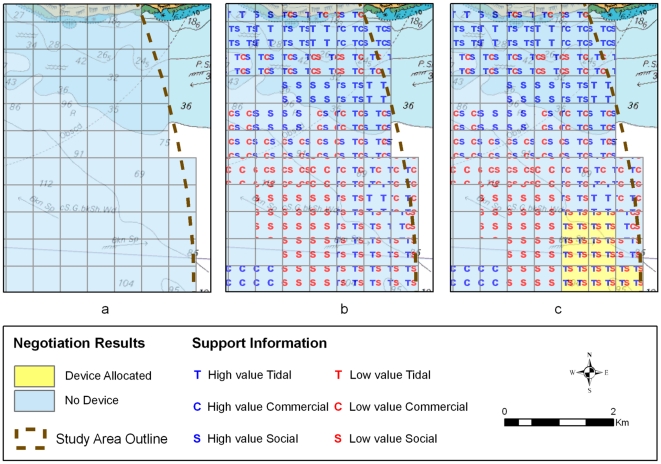
Steps taken during the ‘negotiation’ workshop. a) the underlying nautical chart, b) the best and worst areas for each of three stakeholder groups, c) the negotiated device allocation area.

The aim of the ‘local-knowledge’ workshop was to gather spatially explicit data from local sea-users at relevant spatial scales. This was achieved by showing the participants the GIS layers used to produced the aggregated value maps (Figure 2a). Each participant then used the ‘map valuation tool’ to draw locations of particular significance to their stakeholder group (Figure 2b), and then to re-value the map based on these drawings (Figure 2c). The aim of the ‘negotiation’ workshop was to build a consensus on the best locations for situating potential tidal devices. This workshop made use of the original individual GIS layers; the original stakeholder value maps and the revised stakeholder value maps. In this workshop the participants made use of the ‘MCA trade-off tool’. Based on the spatial MCA values, best and worst areas were indicated on the touch-table using blue and red capital letters respectively for each of three sectors: Tidal (T), Commercial (C), and Social (S). These identified negotiable cells optimal for one stakeholder but not for the other and vice versa. Stakeholders were asked to trade negotiable cell alternatives as follows: two cells (0.25 km^2^: ∼40 MW), five cells (0.25 km^2^: ∼100 MW) and ten cells (0.25 km^2^: ∼200 MW) (Figure 3). Participants in the ‘negotiation’ workshop were further asked to evaluate the data sources upon which the negotiations were based, and to complete a survey questionnaire relating to their background and the tasks they had to perform.

## Results

Results are presented in the context of the identification of potential conflicts, spatial data collection and negotiation support in workshop settings.

### Identification of potential conflicts between users

Conflict between marine users emerged throughout the workshop process. During the ‘local-knowledge’ workshop, the tourism representative suggested that although the area may not be used on a regular basis, when placed in a situation of potential loss of access it is likely that all stakeholders would exaggerate the importance of the area and

*“Suddenly find that half of their income comes from that area”.*

Tourism Representative

During the ‘negotiation’ workshop, it was pointed out by the fishing association representative that

“*the negotiations are based upon an assumption that tidal energy would take precedence over other stakeholders*”.

Fishing Association Representative

It was further suggested that areas further offshore may be preferable for tidal devices, to avoid conflict with other stakeholders, yet concerns were raised that these areas may be too deep for cost effective development.

Suggestions of inter-industry conflict were also noted. During the ‘negotiation’ workshop the fisheries association representative stressed that fishers do not want to share information on fishing practices with each other

“*if all fishermen were brought together around the touch table, it would be impossible to establish important fishing areas within the study site due to potential competition for resources.*” .

Fishing Association Representative

### Collection of spatial data at local scales

**Figure 4:**
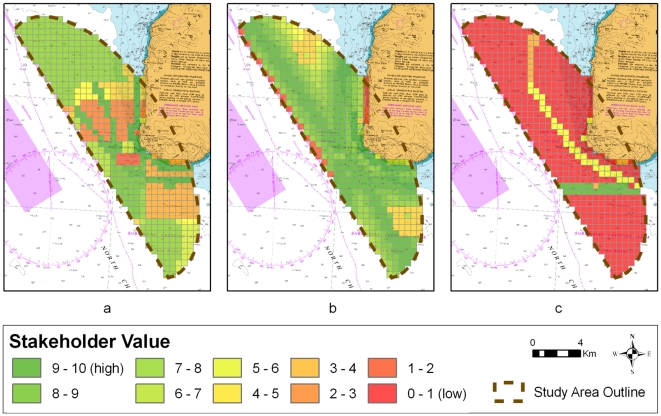
The results of the ‘local knowledge’ workshop. a) fisheries stakeholder output, b) recreational sailing stakeholder output, c) tourism stakeholder output.

Three new stakeholder value maps were generated (Figure 4) during the ‘local-knowledge’ workshop based on the knowledge of the local fisheries, recreational shipping and tourism stakeholders. We were unable to access local-knowledge relating to tidal energy, shipping or the environment.

Creel fishing for crab and lobster, scallop dredging and scallop diving occurred within the area, and these were noted on the map (Figure 4a). There were some areas indicated within the study site where the tides are too strong for fishing including a tidal race and over-falls to the west of the Mull of Kintyre. Two routes for fishing vessel transit around the Mull of Kintyre were identified. These were weather dependent: with boats staying close in to the western shore of the Mull in settled weather and steam further out from the shore in rougher weather. All identified fishing areas were allocated the highest value possible by the local fisheries stakeholder.

Participants added a considerable amount of data to the recreational and yachting value map (Figure 4b). Discussions revealed that the area around the Mull of Kintyre was mainly a passage route for recreational vessels. A route hugging the coast of the peninsula was marked as the most important although also weather-dependent. Recreational boats were said to be limited to the west by the shipping lane with the area just to the east of the shipping lane less used except during bad weather. Recreational and yachting participants found it difficult to give values to cells within the study site. Reasons for this included the fact that sailing around the Mull involved a lot of tacking; poor weather means that routes can change; and depending on the experience of the yachtsman, routes are not always followed closely.

Discussion of the tourism map (Figure 4c) indicated no regular marine tourism around the Mull of Kintyre, leaving much of the study site marked as ‘low value’. The local wildlife tour operator takes tourists mainly to Sanda Island, and has not travelled around the Mull in three years. Other operators in the area include a fast ferry service operating between Campbeltown and Ballycastle through the summer months which passes through the study site. The paddle steamer *Waverley* travels around the Mull twice a year. All local stakeholders agreed that no kayaking occurs within the area due to the currents, and that the dive sites are unpopular.

Discussion in the ‘negotiation’ workshop regarding the quality of the data used led to suggestions that the published tidal data were inaccurate. The fisheries stakeholder also advised that all areas of the proposed site were used either by creel or pelagic fisheries thus contradicting the ‘local-knowledge’ map from the previous workshop. It was suggested that dealing with commercial fishing as a single sector was simplistic and that different types of fishing should be considered. Furthermore, results of the feedback questionnaire indicated that while 40% of participants felt there was sufficient information available to help with negotiation, 60% of participants felt there was insufficient information.

### Support negotiation in stakeholder workshop setting

**Figure 5:**
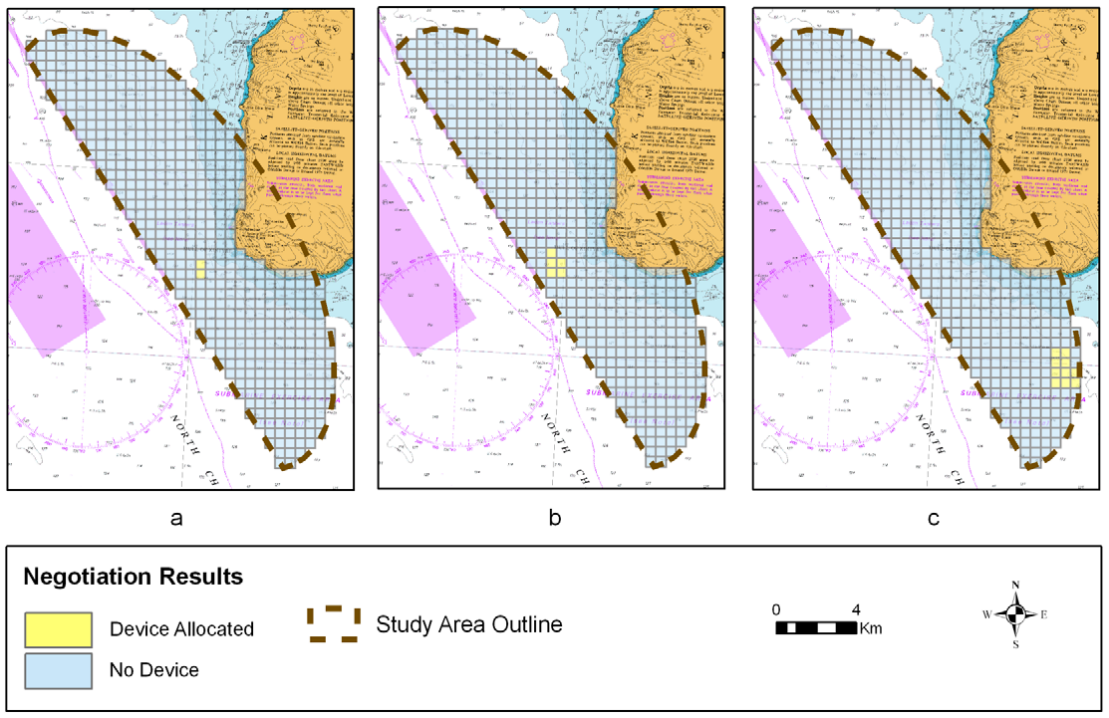
The results of the ‘negotiation’ workshop. a) device allocation for ∼40 MW alternative, b) device allocation for ∼100 MW alternative, c) device allocation for ∼200 MW alternative.

Negotiations were successful with maps created for all device allocation alternatives. (Figure 5). For the ∼40 MW alternative, two cells close to the shipping lane were selected by the participants on the basis of compromise between depth for the device and stakeholder use (Figure 5a).

The ∼100 MW negotiation decision was similar to the results of the ∼40 MW scenario although the selected cells were shifted to the west to allow for tacking of recreational vessels between land and the tidal devices so as to avoid sailing around the devices when in transit. All participants with the exception of the fishing association representative concurred with this allocation of devices (Figure 5b).

As the number of cells for the negotiation scenario was increased to 10 (∼200 MW), the issue of exclusion zones was raised particularly as it related to its impact upon the fishing industry both in terms of access loss and safety implications for vessels (i.e. turbines acting as winches and pulling fishing boats under). Fisheries stakeholders suggested a realistic safe exclusion zone for fishing might be 500 m, similar to those around oil and gas platforms. It was suggested that one large block of cells would lose less space for fisheries than several smaller ones (due to the resulting exclusion zone, which in terms of this study would have a device/exclusion zone ratio of 1∶8 cells, reducing to 1∶5 then 1∶4 etc as the number of cells in a block increase). Nevertheless concerns remained over the possible size of an exclusion zone given the narrowness of some transit areas. The recreational shipping representative suggested that ten cells would interfere with yachting around the Mull of Kintyre if the cells were kept in the same area as previous alternatives. A site further to the south was suggested as a more appropriate location. All stakeholders agreed that the ∼200 MW alternative should be allocated in this part of the study site (Figure 5c).

Results from the feedback questionnaire indicated that 80% of participants found the combination of knowledge from researchers and stakeholders to be most helpful, and that although they had little or no experience of a similar type of technology, 70% of participants did not find the touch table difficult to use. All participants recommended the workshops as a method to address local spatial planning.

## Discussion

This is the first successful demonstration, within the marine environment, of an SDSS approach combining GIS, spatial MCA, a touch-table and stakeholder workshops, reflecting the success of previous demonstrations in the terrestrial arena [34]. The technology combined with the workshop format successfully involved multi-sectoral stakeholders, identified potential conflict and contributed to a broader understanding of the complexity of problems facing different stakeholders. It gathered spatial information on user values for particular areas (with a change in user values between the initial data and final maps used for negotiation) and enabled potentially conflicting parties to negotiate optimal site locations.

Although SDSS is a standard method used in resource management, our particular approach offers more than those previously used. The approach enabled participants to view the “larger picture”. During the ‘local-knowledge’ workshop all contributors participated enhancing interactivity and communication across sectors. Participants shared ideas, asked each other questions, and brought up pertinent points which were then developed by others. This interactivity, which could also be seen during negotiations, is central to this approach and would not occur when using online data gathering methods. Furthermore, the use of spatial MCA allowed individual values for parts of the ecosystem to be traded-off in the absence of measured or estimated ecosystem service values (e.g. amenity values such as yachting) overcoming what is often seen as a barrier to implementation of the EA [9].

In concurrence with existing literature [9], [10], the workshops identified problems with existing baseline data, suggesting a need for improved data generation at relevant spatial scales. This can be supported by use of the ‘map valuation tool’ which facilitated integration of local-knowledge within negotiations. The question of scale is highly significant in stakeholder negotiation. Data collection at a relevant scale for development is imperative and tools such as the ‘map valuation tool’ are a method of cost-effective data collection. Whilst some data can only be collected commercially e.g. bathymetry and tidal flows, other data is better provided by local people particularly on a fine-scale e.g. seabed-type and stakeholder use, ideally across all relevant stakeholder groups. Local knowledge cross-referenced with commercially gathered data could enhance robustness and reliability. Including local-knowledge in resource management is becoming increasingly important, and it has been suggested that even if there is the desire to use non-scientific knowledge, it is often not known how [38]. The approach used in this study allows for the collection, collation and integration of local-knowledge with scientific data; and furthermore is a form of ‘joint fact-finding’ [39] central to consensus building and dispute resolution.

The workshops revealed areas of conflict and our aim to develop a consensus raised several points for consideration. Offshore development may effectively place particular areas of the common sea space under private control and place spatial restrictions on many ongoing activities. This may result from exclusion zones as suggested within the workshops or from regulatory guidance [40]. Avoidance of installation areas may mean that commercial practices such as fishing can no longer take place within installation sites, and may lead to increased steaming time for all vessels using the area, increased costs for all concerned, and may fundamentally change social dynamics. Fishers, in particular, are perceived to be in a weak position due to the impetus of international policy driving offshore renewable energy [41] and the trend toward more sustainable fisheries particularly in European Marine policies [11]. The fishing industry is likely to suffer the largest impact from offshore energy. Substantial regulation such as Total Allowable Catch (TACs), gear regulations, closed seasons, closed areas, minimum allowable sizes and limits to time spent at sea [42] has meant that access to commercial fishing is already being reduced. This may be exacerbated with the inclusion of marine energy installations. These effects will clearly be site specific and thus should be addressed on a case-by-case basis.

The remark that fishers' would be unlikely to reveal information on fishing site locations in a public arena indicates the personal value of spatial information to fishers in a quickly reducing marine area. Fisher knowledge is a professional asset which is used not only for catching fish, but also for regulating relationships within the industry [43], and reluctance on the part of the fishers to provide the information necessary for successful negotiation could result in inequitable representation and consequently further declines in fishing activity. Furthermore, the suggestion that even in areas of low use, when placed in a situation of possible loss of access it is likely that all stakeholders would exaggerate the importance of that area leads to the question: is sea-use conflict based upon the need to protect income and therefore personal security? In a study of the implications of offshore wind farm developments on fishermen, a major concern was the loss of traditional fishing grounds and consequently the loss of future livelihoods [44]. Our workshops supported this position.

The suggestion that the tidal energy industry takes precedence over any other stakeholder may be a reason for potential conflict between developers and existing users. Scottish government renewable energy targets and the proactive approach taken to achieve them (e.g. the Saltire prize [45]) suggest that renewable energy development is high on their agenda and UK policy has previously given precedence to allocating offshore wind farm leasing areas ahead of other considerations such as Natura 2000 sites [46]. Priorities for use of the marine environment will be set by governments in the context of their obligations under national and international law, as well as their economic and social targets and, ultimately, it is they who will decide whether renewable energy takes precedence over existing marine activities. Previous consequences of the privatisation of coastal areas have included the worsening of economic conditions in coastal communities, the increased exploitation and degradation of remaining accessible resources, the displacement of communities, and conflicts between stakeholders [47].

Furthermore, the proposed need for exclusion zones for safety reasons raises the question of liability. The potential for export from restricted areas [48], [49] may lead fishermen to ‘push’ the boundaries of exclusion zones, particularly under economic stress (i.e. now, in the recession). There are currently no known guidelines concerning the identification of liability and insurance costs in the case of accidents involving renewable devices and this should resolved prior to device installation.

Overall, the approach addressed the barriers to implementing EA theory in practice: using a multi-sectoral focus, valuing often unquantifiable ecosystem services and enabling the public to participate in the EA process. It also addresses two GEnS programmes of measures: the use of spatial controls (MSP), and stakeholder participation. The value-map approach is not only low cost, but also creates support for the data and (potentially) re-enfranchises disaffected stakeholders. The results from the negotiation were promising: stakeholders with opposing perspectives successfully managed to identify areas where tidal devices might be situated with minimal disruption to existing activities and, furthermore, the offshore industry has expressed interest in the approach.

For marine spatial planning to be undertaken within the context of the EA, in a way which is equitable to all stakeholders, the authors would make the following recommendations:

- Data collection at the relevant scale is essential, and anecdotal and survey-based data cross-referenced to increase robustness and reliability.
- The issues of who/what takes precedence, ownership and rights in the marine space and liability must be addressed by policymakers/regulators.
- The combination of touch-table, GIS, MCA and stakeholder workshops is ideally suited to facilitating the EA to marine management and we recommend its implementation by those dealing with MSP.

## Supporting Information

Table S1 **GIS data sets aggregated by stakeholder group.**
(DOC)
