# Supplementary material for: Interactive Marine Spatial Planning: Siting Tidal Energy Arrays around the Mull of Kintyre
Source: PLoS One. 2012 Jan 11;7(1):e30031. doi: 10.1371/journal.pone.0030031 (PMC3256193; doi:10.1371/journal.pone.0030031)
Supplement: Supplementary file 1 [file pone.0030031.s001.doc]

Data Type	Data Set	New or Existing Shapefile	Type	Source	Original Aims	Date	Collection/ Capture
Commercial	IMO Tanker Routes	Existing	Vector	Marine Scotland	IMO Agreed Tanker routes in Scotland		
Commercial	Ports	New	Vector	Ports of Scotland		Jun-10	Not all ports included. Ports chosen according to: capabilities (max length and draft – based on barges used by MCT for Seagen), and being developed according to SE NRIP
Cultural	Wrecks	Existing	Vector	EDINA	British Ordnance Survey Maps	Jan-08	Based on re-engineered data captured by UKHO from paper charts for the production of ENCs (Electronic Navigational Charts)
Cultural	Scheduled Monuments	Existing	Vector	Historic Scotland	To allow the user to identfiy the approximate position, size and extent of scheduled monuments in Scotland.	May-08	
Environmental	Scot SSSIs	Existing	Vector	SNH	As above.	Jan-10	
Environmental	Cetaceans	New	Raster	JNCC	Atlas of Cetacean distribution	2003	Dataset created from spotting co-ordinates. Data came from JNCC - mix of SCANS, Seabirds at Sea Team and UK Mammal Society Cetacean Group (now Seawatch Foundation).
Environmental	Seabirds	New	Raster	JNCC - Obis Seamap	Biogeographic Information System Spatial Ecological Analysis of Megavertebrate Populations, is a spatially referenced online database.		Seabirds at Sea Team (SAST) has conducted numerous surveys of seabird and cetaceans at sea from ships and aircraft using standard methods
Physical	UK Seabed	Existing	Raster	MESH	For display on UKSeaMap webGIS and download	Mar-10	UK datasets compiled from a number of studies including offshore windfarm, aggregate, oil & gas EIA's; BGS Surveys; and other research projects - full list of datasets at http://www.searchmesh.net/default.aspx?page=1403&country=GB
Physical	Tidal resource	Existing	Raster	BERR	Atlas of UK Marine Renewable Energy Resources project.	Mar-08	Tidal data supplied by Proudman Oceanographic Laboratory (POL) on behalf of BERR for the Atlas of UK Marine Renewable Energy Resources project.
Physical	Depth Data	Existing	Raster	EDINA	British Ordnance Survey Maps	Jan-08	Based on re-engineered data captured by UKHO from paper charts for the production of ENCs (Electronic Navigational Charts)
Recreation	RYA Cruising Routes	Existing	Vector	RYA	UK Coastal Atlas of Recreational Boating	2008	Data compiled initially from existing sources and translating the information onto the RYA's Geographical Information System (MapInfo) with comments on the maps from stakeholders, additional routes identified and some routes removed or redefined
Fishing	Fishing Satellite Data	Existing	Raster	DECC	Produced by Anatec UK Ltd for the Maritime Data project	2007	Information on Fishing Satellite Data for UK waters, which covers fishing vessels of overall length 15m and over. The data only covers UK-registered vessels.
Fishing	Fishing Surveillance Data	Existing	Raster	DECC	Produced by Anatec UK Ltd for the Maritime Data project	2008	The results of analysis of fishing surveillance (sightings and patrols) in UK waters between 2006 - 2008
Fishing	Fishing Value	Existing	Raster	APBMer	Create a series of consistent information layers relating to the distribution and economic value of commercial fishing and shellfisheries in UK waters	Mar-09	The layers display the annual mean Fish Value for years 2004 to 2007. The data was created from the following datasets - processed VMS data from Cefas and FRS and financial landing value data from MFA.
Commercial	Commercial Shipping	Existing	Raster	National Center for Ecological Analysis and Synthesis	To estimate commercial shipping activity	Feb-08	Data collected from 12 months beginning October 2004 (collected as part of the World Meteorological Organization Voluntary Observing Ships Scheme
